# Wilson Disease Hiding in Plain Sight: A Case Report of Psychosis and Catatonia Revealing Underlying Liver Dysfunction

**DOI:** 10.3390/reports8040261

**Published:** 2025-12-11

**Authors:** Adela Georgiana Buciuc, Vanessa Padilla, Dante Durand, Espinel Zelde

**Affiliations:** 1 Department of Psychiatry and Behavioral Sciences, University of Miami/Jackson Health System, Miami, FL 33101, USA; 2 Department of Psychiatry and Behavioral Sciences, University of Miami Miller School of Medicine, Miami, FL 33136, USA

**Keywords:** Wilson disease, psychosis, catatonia, copper metabolism, liver transplantation

## Abstract

**Background and Clinical Significance**: Wilson disease is a rare autosomal recessive disorder of copper metabolism that can initially present with psychiatric symptoms, leading to delays in accurate diagnosis and treatment. Adult-onset cases may be misdiagnosed as primary psychiatric disorders, particularly when hepatic signs are subtle or absent. Early recognition is critical to prevent irreversible neurological and hepatic damage. **Case Presentation**: A 48-year-old Hispanic male developed persecutory delusions, cognitive decline, and ultimately catatonia over a three-year period. He was initially diagnosed with a primary psychiatric disorder and treated with antipsychotics, which caused severe extrapyramidal side effects. Further evaluation revealed markedly abnormal liver function tests, low serum ceruloplasmin, and elevated 24 h urinary copper excretion. Brain MRI showed characteristic findings of Wilson disease, and liver biopsy confirmed the diagnosis. The patient was started on trientine and zinc sulfate, but progressive hepatic dysfunction necessitated liver transplantation. Following a successful transplant, the patient experienced significant neurological and psychiatric recovery. **Conclusions**: This case underscores the importance of considering Wilson disease in patients presenting with atypical or treatment-resistant psychiatric symptoms, particularly when accompanied by abnormal liver function or intolerance to antipsychotics. Timely, multidisciplinary evaluation is essential to avoid misdiagnosis and initiate appropriate therapy. Early intervention can significantly improve both psychiatric and medical outcomes in Wilson disease.

## 1. Introduction and Clinical Significance

Wilson disease is an autosomal recessive condition of copper metabolism caused by mutations in the *ATP7B* gene, which encodes a copper-transporting protein [1]. The defective *ATP7B* protein leads to decreased incorporation of copper into apoceruloplasmin as well as decreased biliary excretion of copper. The excessive copper accumulates in the liver, brain, and other organs [2,3]. A wide range of psychiatric manifestations have been reported in Wilson disease, including psychosis, depression, mania, behavioral problems, neurocognitive impairment, and personality changes [4]. Psychiatric symptoms may obscure the underlying etiology and delay the appropriate diagnosis, particularly when liver dysfunction is subclinical. Catatonia, although rare in Wilson disease, has been described in case literature and poses additional diagnostic complexity [5]. Here, we present the case of an adult immigrant male whose presentation with psychosis and catatonia led to the diagnosis of Wilson disease, underscoring the need for heightened clinical suspicion in similar scenarios.

## 2. Case Presentation

### 2.1. Case Report

We present the case of a 48-year-old Hispanic man, a college graduate, with recent diagnosis of unspecified psychotic disorder and medical diagnosis of liver hemangiomas, hypothyroidism and hyperlipidemia who had recently immigrated to the United States with his family. He was unemployed and uninsured at the time of presentation. The patient arrived at the emergency department of a large urban medical center following two days of decreased food intake, generalized weakness, poor sleep, and significantly diminished verbal communication with his family. The patient was initially admitted to the Internal Medicine service. Two days into his medical hospitalization, he was evaluated by the Consultation-Liaison Psychiatry team due to concern for depressive symptoms.

The patient’s medical and psychiatric history was obtained primarily from his family, as he remained minimally communicative during the initial evaluation. He grew up in an urban area in South America, and his family history was notable for consanguinity; his father and two paternal uncles had each married one of three sisters who were first cousins. Among these households, each family has had one child who has developed severe or fatal liver disease. In addition, a male cousin had died in his 50s from cirrhosis, and a female cousin died in her 20s during the postpartum period from autoimmune hepatitis.

The patient had been psychiatrically and medically healthy until the age of 45, when, while still residing in South America, he began experiencing progressive fatigue, psychomotor slowing, cognitive difficulties, and an unintentional weight loss of nine kilograms over a three-month period. According to his wife, he also developed vague paranoid ideation related to his work environment. Initial laboratory testing revealed elevated iron saturation and iron levels, prompting a presumptive diagnosis of hemochromatosis; however, this was later ruled out due to negative homeostatic iron regulator protein (HFE) gene testing. Imaging revealed liver hemangiomas, and a liver biopsy was deferred due to the risk associated with those lesions. His medical symptoms partially improved without further intervention, and no additional workup was pursued at that time.

Over the following two years, the patient became increasingly suspicious, guarded, and anxious. During this period, he immigrated to the United States with his family. Approximately four months after his arrival, he began to exhibit well-formed persecutory delusions involving multiple distinct themes. He believed that a family member was sabotaging his employment and attempting to have him terminated. He also became convinced that an intelligence agency was monitoring his activities, tapping his phone, and planning to incarcerate him. Additionally, he expressed the belief that his food was being adulterated with coloring agents designed to track him, leading to intermittent food refusal. Despite possessing legal immigration status, he was persistently preoccupied with the belief that he would be arrested by immigration authorities. He presented to a community mental health clinic, where he was diagnosed with an unspecified psychotic disorder. Laboratory testing revealed abnormal liver enzymes: AST 199 U/L (normal: 10–40 U/L), ALT 193 U/L (normal: 7–56), and alkaline phosphatase 241 U/L (normal: 44–147). He was started on olanzapine 5 mg daily, which partially reduced his psychotic symptoms but led to severe extrapyramidal side effects including laryngeal spasm, limb dystonia, and generalized rigidity. The antipsychotic was discontinued.

Several months later, the patient was admitted to a large urban medical center. On evaluation by the consultation-liaison psychiatry team, the patient exhibited jaundice, scleral icterus, and lower extremity edema. He was alert and oriented to person, place and time. His grooming was adequate, and he appeared his stated age. He exhibited immobility, sitting abnormally still and only interacting briefly with the interviewer. Neurological examination did not reveal any focal neurological signs. He displayed mutism, speaking less than 20 words in 5 min, and responded with single-word answers. He denied symptoms of depression but his affect was blunted. No psychotic behavior was observed. Other catatonic signs included poor eye contact, decreased blinking, negativism, ambitendency, posturing, and resistance to passive movements (gegenhalten) and autonomic instability (tachycardia). Although formal documentation using the Bush-Francis Catatonia Rating Scale (BFCRS) was not available in the electronic medical record, the patient met DSM-5-TR criteria for catatonia, with more than three characteristic features. He was given a lorazepam challenge (1 mg intravenous) and after 5 min, the patient’s catatonic symptoms improved and he no longer displayed rigidity, autonomic abnormalities, and was able to move spontaneously. Although he spoke softly with a low tone of voice, he was able to respond appropriately using full sentences. He was able to drink and eat without difficulty.

Laboratory results revealed total bilirubin 7.3 mg/dL (normal: 0.1–1.2), direct bilirubin 5.7 mg/dL (normal 0.0–0.3 mg/dL), AST 128 U/L, ALT 123 U/L, and alkaline phosphatase 639 U/L. Serum ceruloplasmin was low at 14.5 mg/dL (normal 20–35 mg/dL), and 24 h urinary copper was elevated at 254 mcg/24 h (normal < 40–60 µg/24 h). MRI of the brain showed T1 hyperintensity in the basal ganglia and ventrolateral thalami bilaterally, with sparing of the globus pallidus. Ophthalmologic evaluation did not reveal Kayser-Fleischer rings. Abdominal ultrasound demonstrated a cirrhotic liver with multiple hyperechoic lesions. Liver biopsy revealed nodular architecture with parenchymal collapse, cholestasis, bile plugs, and lobular inflammation. Histological features suggestive of Wilson disease included centrilobular necrosis, and mild macrovesicular steatosis. Prussian blue staining was negative for iron, supporting a non-hemochromatosis etiology. Rhodamine staining was positive for copper. Quantitative hepatic copper concentration was not measured. Genetic testing for ATP7B was not performed due to institutional resource limitations at the time of diagnosis.

Based on the Leipzig scoring system [6], the patient scored 6 points: low ceruloplasmin (1 point), elevated urinary copper (2 points), neurological signs (1 point), and compatible liver histology (2 points), confirming a diagnosis of Wilson disease. The patient was initiated on trientine 250 mg three times daily and zinc sulfate 220 mg three times daily based on gastroenterology consultation. He was evaluated by the transplant hepatology service and placed on a liver transplant waitlist. The psychiatry team recommended starting lorazepam 0.5 mg oral twice a day for catatonia symptoms, with good response and tolerability during hospitalization. Follow-up with psychiatry and neurology was recommended.

At a three-month follow-up, the patient continued to experience residual blunted affect, postural instability, and tremors. He remained adherent to trientine and zinc therapy. However, due to lack of insurance, he did not follow up with psychiatry or neurology services. At the time of manuscript submission, the patient had undergone successful liver transplantation and was in stable condition.

### 2.2. Strengths and Limitations of the Clinical Approach

This case underscores the diagnostic challenges and clinical implications of Wilson disease presenting primarily with psychiatric symptoms. The patient’s progression—from cognitive slowing and mild paranoia to florid psychosis and catatonia—demonstrates how metabolic disorders, particularly inborn errors of metabolism (IEMs), can closely mimic primary psychiatric conditions, often resulting in delayed diagnosis and inappropriate treatment [7,8,9]. The overlap in symptomatology is well-documented: for example, Wilson disease, urea cycle disorders, porphyrias, and Niemann-Pick disease type C can present with psychosis, mood disorders, or cognitive changes, and are frequently misdiagnosed as primary psychiatric illnesses, particularly when neurological or systemic signs are subtle or absent [10,11]. A notable strength in this case was the consultation-liaison psychiatry team’s prompt recognition of catatonia and use of a lorazepam challenge, which served both diagnostic and therapeutic purposes. The team’s decision to avoid further neuroleptic use following severe extrapyramidal side effects also reflects prudent clinical judgment, in line with evidence highlighting increased neuroleptic sensitivity in Wilson disease [12]. Subsequent interdisciplinary collaboration facilitated a timely and comprehensive diagnostic workup, including neuroimaging and liver biopsy.

However, the case also reflects several limitations. Diagnostic delay occurred despite early indicators such as abnormal liver enzymes, mid-life neuropsychiatric symptoms, and a family history of liver disease. While initial evaluation for hemochromatosis was appropriate, a broader differential diagnosis was not pursued after negative HFE gene testing. The absence of quantitative hepatic copper measurement represents a further limitation, as this remains a key diagnostic criterion. The BFCRS, a 23-item clinician-rated scale widely used in general medical settings, is typically employed to assess severity and monitor treatment response, and may have provided further objective data had it been formally applied in this case [13,14]. Additionally, limited access to care and a lack of continuity in outpatient follow-up, largely due to the patient’s uninsured status, likely contributed to delays in definitive treatment and comprehensive management.

## 3. Discussion

Wilson disease exhibits a heterogeneous clinical presentation, with hepatic and neuropsychiatric manifestations that may occur independently or in varying combinations, often complicating early diagnosis, particularly in the absence of classic features [15,16]. Hepatic manifestations often appear earlier and range from mild liver enzyme abnormalities to cirrhosis or, less commonly, acute liver failure [15]. Neurological symptoms can include movement disorders such as tremor, dystonia, Parkinsonian rigidity, dysarthria, and pseudobulbar signs [15]. Psychiatric presentations are diverse, with depression being most common, but bipolar disorder, psychosis, anxiety, and behavioral changes are also reported [15,16]. Kayser–Fleischer rings are a classic ophthalmologic sign but are not universally present [15].

Dysregulation of hepatic function plays a central role in the pathogenesis of neurological manifestations in Wilson disease [15,16,17]. Hepatic copper overload impairs systemic copper homeostasis, leading to copper deposition in neural tissue—especially in the basal ganglia, where it promotes oxidative stress, mitochondrial dysfunction, and neuroinflammation [17]. These mechanisms contribute to the psychiatric and motor symptoms observed in many patients, and highlight the importance of early hepatic intervention to prevent or mitigate neurological sequelae [17].

The review emphasizes that Wilson disease can present at any age and may mimic other hepatic, neurologic, or psychiatric conditions, justifying its reputation as a “clinical chameleon” [15]. This broad phenotypic variability underpins the need for a high index of suspicion and comprehensive diagnostic evaluation in patients with unexplained liver, neuropsychiatric, or multisystem disease. Untreated Wilson disease is fatal, underscoring the importance of timely diagnosis. The American Association for the Study of Liver Diseases (AASLD) states that prior to the advent of chelation therapy, Wilson disease was universally lethal, and survival into adulthood was rare [16].

Our patient presented with several features that pointed toward a psychotic disorder due to a medical condition: abnormal liver function tests, increased sensitivity to extrapyramidal symptoms (EPS) with atypical antipsychotics, and catatonia. The patient initially exhibited psychotic symptoms that improved with olanzapine, which had to be discontinued due to EPS. After stopping the antipsychotic, the patient’s illness progressed, and eventually, he presented with catatonic features.

Catatonia in medical patients is most commonly classified as catatonia due to another medical condition (CDGMC). Multiple validated instruments exist to assess catatonia, including the DSM-5-TR diagnostic criteria [18], the BFCRS [13,19], and the KANNER scale which is often used in neurodevelopmental and autism spectrum contexts [20,21]. While our diagnosis relied primarily on clinical features and DSM-5-TR criteria, the recent literature has emphasized the need for standardized assessments, as DSM-5 criteria alone may under-detect catatonia [19].

Over 50% of catatonia cases in medical settings are attributable to general medical conditions, with Wilson disease representing a rare but important etiology [22]. Tools such as the MINDSET mnemonic have been proposed to systematically classify the broad range of CDGMC etiologies and may be useful in future case-based teaching and documentation [22].

Current clinical guidelines emphasize the importance of promptly considering Wilson disease in middle-aged adults without prior psychiatric history who present with psychosis and cognitive decline, particularly when accompanied by abnormal liver enzymes and extrapyramidal features [15]. AASLD specifically highlights that psychiatric manifestations, including psychosis and cognitive impairment, are common and may precede or accompany hepatic and neurological findings [16]. Notably, up to two-thirds of patients have psychiatric symptoms at disease onset, and cognitive deficits may be subtle or only detectable with formal neuropsychological testing, especially in those with hepatic or neurologic involvement [16].

Evaluation of patients in which Wilson disease is suspected should include a physical examination for evidence of chronic liver disease, baseline liver function tests, ophthalmologic evaluation for Kayser–Fleischer rings, and assessment of family history of liver or neuropsychiatric disease [4]. However, as occurred in this case, the absence of Kayser-Fleischer rings does not exclude the diagnosis of Wilson’s disease [1,4].

Our patient responded to a lorazepam challenge, confirming catatonia, a rare but treatable manifestation of Wilson disease. The use of antipsychotics in such patients is often limited by hypersensitivity to extrapyramidal effects [12]. While olanzapine improved psychotic symptoms, it triggered severe dystonia, necessitating its discontinuation. This sensitivity is attributed to underlying basal ganglia dysfunction from copper deposition, which increases vulnerability to the motor side effects of antipsychotics [23,24]. Copper toxicity leads to neuronal apoptosis, glial activation, and oxidative stress within the corpus striatum and other basal ganglia structures, which are critical for motor control [23].

Initial management of Wilson disease includes dietary modifications, immunizations, abstaining from alcohol, and specialty referrals [1]. Patients are advised to avoid foods high in copper, such as shellfish and chocolate, and to take precautions with water sources if consuming well water [1]. Immunizations for hepatitis A and B are recommended, along with vaccines for liver disease [25]. Avoiding alcohol, especially heavy use, is crucial [25]. Specialty referrals to a hepatologist, neurologist, psychiatrist, dietician, and genetic counselor are advised [15]. Initial therapy is based on symptom severity; symptomatic patients typically receive copper chelators like D-penicillamine or trientine, while asymptomatic patients may be managed with lower-dose chelation or zinc therapy [1]. Chelators are generally effective, with D-penicillamine and trientine showing similar efficacy in symptom improvement [1]. Trientine and zinc were well tolerated in this case. According to guidelines from AASLD and the European Association for the Study of the Liver–European Reference Network (EASL-ERN), copper chelators are recommended as first-line therapy for patients with significant hepatic involvement [16,26,27]. However, in cases of severe liver dysfunction requiring rapid copper reduction, combination regimens may be considered as an alternative strategy [16,26,27]. The patient’s positive response to treatment, despite diagnostic delays, illustrates the potential for recovery when appropriate care is initiated. If psychiatric symptoms do not resolve with copper chelation alone, antipsychotic therapy may be necessary, but the risk of severe motor side effects should always be considered. The literature consistently emphasizes the need for individualized dosing and close monitoring for neuroleptic-induced movement disorders in this population [16,28].

## 4. Conclusions

This case highlights the importance of including Wilson disease in the differential diagnosis of atypical psychiatric presentations, especially when accompanied by hepatic abnormalities or antipsychotic sensitivity. A thorough diagnostic evaluation, encompassing serum ceruloplasmin, 24 h urinary copper, liver biopsy, and neuroimaging, remains crucial, even in the absence of hallmark features such as Kayser-Fleischer rings. Timely identification and initiation of treatment are essential to halt disease progression and improve prognosis. Effective management of such complex cases relies on coordinated interdisciplinary care among psychiatry, neurology, hepatology, and transplant services.

## Data Availability

The original contributions presented in this study are included in the article. Further inquiries can be directed to the corresponding author.
